# Predicting nutritional status during pregnancy by women's empowerment in West Shewa Zone, Ethiopia

**DOI:** 10.3389/fgwh.2023.1147192

**Published:** 2023-06-19

**Authors:** Tizita Dengia Etea, Alemayehu Worku Yalew, Mitike Molla Sisay, Solomon Shiferaw

**Affiliations:** ^1^ Department of Public Health, Ambo University, Ambo, Ethiopia; ^2^ School of Public Health, College of Health Sciences, Addis Ababa University, Addis Ababa, Ethiopia

**Keywords:** women’s empowerment, nutritional status, pregnant women, West Shewa, Ethiopia

## Abstract

**Background:**

Considerable proportions of pregnant women are affected by poor nutrition outcomes in Ethiopia. Women's empowerment, on the other hand, is highly recognized as a means to achieve better maternal nutrition outcomes. However, the role of pregnant women's empowerment in nutritional status during pregnancy has not been empirically examined in Ethiopia. This study aimed to address this gap.

**Objective:**

To assess the association of individual and composite women's empowerment dimensions with pregnant women's nutrition outcomes in West Shewa Zone, Ethiopia.

**Methods:**

A health facility-based cross-sectional study was performed on 1,453 pregnant women living in West Shewa Zone, Ethiopia, in 2021. Exploratory and confirmatory factor analyses were conducted on half of the samples to identify and validate dimensions of pregnant women's empowerment. The associations between pregnant women's empowerment dimensions and anemia status and mid upper arm circumference levels were examined by logistic regressions.

**Results:**

Composite pregnant women's empowerment was positively associated with both anemia status and mid-upper-arm circumference level. The odds of not being anemic were higher among pregnant women empowered in economic [adjusted odds ratio (AOR) = 1.7, 95% confidence interval (CI): 1.26, 2.22] and assertiveness (AOR = 1.9, 95% CI: 1.46, 2.38) dimensions than those not empowered in these dimensions. Empowered pregnant women in household decision-making (AOR = 1.6, 95% CI: 1.19, 2.22) and psychological (AOR = 1.4, 95% CI: 1.04, 1.85) dimensions had higher odds of having normal mid-upper-arm circumference measures than those not empowered in the respective dimensions. Communication and time dimensions were not significantly associated with any of the nutrition outcomes.

**Conclusions:**

This study suggests that empowered pregnant women are nutritionally better off than their less empowered counterparts. This is also important in child health outcomes. Policies and programs that aim to improve maternal and child health in the study area need to consider interventions that promote the decision-making power, economic, psychological, and assertiveness dimensions of pregnant women.

## 1. Introduction

Adequate nutrition during pregnancy is an important factor for the health of the mother and the health, growth, and development of the fetus (1–3). Maternal nutrition may affect not only the immediate pregnancy outcomes but also the long-term health of infants, including a higher risk of hypertension in adults (4) and stunting in those born with a low birth weight (5). Even though it is preventable, poor nutritional status affects millions of pregnant women around the world (6). Anthropometric, biochemical, and dietary assessments in developing countries reveal that pregnant women are affected by poor nutritional status (7–9). For instance, the prevalence of anemia among pregnant women has decreased slightly over the years in low- and middle-income countries. In fact, around 40.1% of the pregnant women in these countries are anemic (10). According to the World Health Organization (WHO), anemia, as measured by hemoglobin concentration, is considered to be a public health problem if population studies find an anemia prevalence of 5.0% or higher (11), and WHO has planned to reduce anemia by 50% by 2025 (12).

The National Nutrition Strategy designed by the Ethiopian Government prioritizes improving pregnant women's nutritional status (13). Despite the efforts to address undernutrition at the national level, Ethiopia has a significant proportion of malnourished pregnant women. For instance, the 2016 Ethiopian Demographic and Health Survey (DHS) result shows that 29.1% of Ethiopian pregnant women were anemic (14). The findings in different regions of Ethiopia also confirm that anemia based on hemoglobin measurement during pregnancy is a public health problem (8, 15–17). Poor birth outcomes and adverse maternal outcomes such as preterm birth, stillbirth, small-for-gestational-age, postpartum hemorrhage, and preeclampsia have been associated with low maternal hemoglobin (<11 g/dL) during pregnancy (2). Moreover, pieces of evidence from different regions of Ethiopia indicate undernourishment, which is measured based on mid-upper circumference (MUAC) measurement <23 cm, affecting a large number of pregnant women in the country (7, 9, 18).

Women's empowerment is the process by which women gain greater control over the circumstances of their lives (19). It is underlined that the three dimensions of resources, agency, and achievements should be indivisible in measuring women's empowerment (20). On the other hand, economic, sociocultural, familial/interpersonal, legal, political, and psychological dimensions have been identified as factors that affect women's empowerment (21). The various dimensions and indicators of women's empowerment stress women's empowerment are reflected in their decision-making power, ability to access and control resources, sense of self-worth, and power to control their own and family lives (20–22). Besides being an end goal by itself (23), women's empowerment is considered a means to achieve other important development outcomes such as improvements in maternal nutritional status (24–26). The different dimensions of women's empowerment are reported to affect their nutritional status differently. For instance, a study in the Kalal’e District of northern Benin found a positive association between women's composite empowerment and nutritional status of women. In this study, the leadership dimension was significantly associated with nutritional status measured by body mass index, while decision-making, mobility, economic security, and male involvement in housework did not show a significant association with women's nutritional status (27). In Ethiopia, women with low decision-making autonomy were more likely to be malnourished than those with high decision-making autonomy (28). In another Ethiopian study, which measured women's empowerment using Women's Empowerment in Agriculture Index, control over income and the number of work hours were positively related to women's nutritional status (29).

Most studies in Ethiopia have addressed the determinants of nutritional status of pregnant women from sociodemographic and dietary practice perspectives (7–9, 15–18). However, the role of women's empowerment in pregnant women's nutritional status has not been empirically examined in Ethiopia. Furthermore, the focus on women's empowerment at this particular period lies not only in the concern for the mother's health but also at the heart of newborn's lifelong wellbeing, which is highly dependent on the mother's wellbeing during pregnancy. Also, the dimensions of empowerment give useful information when analyzing associations and setting targets for possible intervention programs to improve health outcomes (30). Thus, it is essential to identify the setting-specific and holistic dimensions of pregnant women's empowerment that are more significant regarding their nutritional status since they are poorly understood in the study area and it is unclear to what extent they contribute to the study's outcomes. This study, therefore, aimed to investigate association between pregnant women's empowerment and their nutritional status.

## 2. Materials and methods

### 2.1. Study design, period, and setting

A health facility-based cross-sectional study was conducted at public health facilities found in West Shewa Zone, Ethiopia, between January and June 2021. According to West Shewa Zone Health Bureau, the zone had nine hospitals and 93 health centers with an estimated total population of 2,869,314.

### 2.2. Study participants

The study participants were randomly selected pregnant women with gestational age between 26 and 34 weeks attending antenatal care (ANC) follow-up at randomly selected public health facilities found in West Shewa Zone during the study period. Assessment in six public health centers and discussions with the health professionals working in the ANC unit in the study area showed that most pregnant women tended to have one or two ANC visits and come for delivery. In addition, most ANC visits occurred during the second and third trimesters. Conversely, hemoglobin concentrations decline in the second trimester and start to increase in the third trimester (11). Therefore, the gestational age between 26 and 34 weeks was chosen to reduce the inclusion of a large number of pregnant women with low hemoglobin levels because of the trimester they are in and to get the required sample size within a relatively shorter period of time. Pregnant women <18 years old, without a partner, and unable to provide valid information because of mental problems and had been living in the study area for less than 6 months before the survey were excluded.

### 2.3. Sample size determination and sampling procedure

This study is part of a prospective follow-up study that assessed the effect of women's empowerment on nutritional status and birth weight among pregnant women attending ANC at public health facilities found in the West Shewa Zone, Ethiopia. Hence, all the pregnant women who participated in the determination of empowerment level among pregnant women were included in this study. The sample size was determined using the single population proportion formula. There was no previous similar study on pregnant women's empowerment status in Ethiopia. Therefore, we took 29.1% (31) as the prevalence of women's decision-making power as one variable from a Bangladesh study to calculate the sample size for this objective. In addition, a 95% confidence interval (CI) (two-sided *α* = 0.05), a 3% margin error, a 1.5% design effect (owing to multistage sampling use), and a 10% non-response rate were considered. The final total sample size was 1,453 pregnant women. The adequacy of the sample size for assessing associations was checked.

Seven districts were randomly selected from the 22 districts found in the West Shewa Zone. A total of 12 government health centers were proportionally selected using a lottery method by considering the number of public health facilities in each district. The total sample size was distributed proportionally based on the average number of second- and third-trimester pregnant women who presented for ANC follow-up in the last 3 months in each health center. Finally, data were collected from every second willing pregnant woman until the required sample size (*n* = 1,453) was met. Data were collected after the women had received ANC services. Public health facilities that did not provide hemoglobin tests and MUAC measurements were excluded from the study.

### 2.4. Data collection

A structured questionnaire was administered face to face by interviewers to collect information on respondents' socioeconomic and demographic factors, obstetric characteristics, and women's empowerment-related data. The questionnaire was initially prepared in English (Supplementary Table S1) and translated into the local language Afan Oromo. Data were collected by women who had completed grade 12 and were trained on the tools and procedures of data collection by the principal investigator. To ensure the validity and reliability of the questionnaire, a pretest was conducted on 30 pregnant women found in one of the districts in the study area but which was not included in the study. Amendments were done on confusing words, sentences, and questions based on the pretest's findings. Qualified and trained midwives and/or nurses working permanently at the antenatal care service provision unit at the selected health facilities were used to inform and link those women who fulfilled the inclusion criteria to the data collectors. In addition, they were responsible for collecting data on the MUAC of pregnant women. Experienced medical laboratory technologists working at the selected health centers took blood samples and conducted hemoglobin tests based on the standard operating procedures (32).

### 2.5. Variables

#### 2.5.1. Dependent variables

The nutritional status of pregnant women, which was determined by their hemoglobin and MUAC levels, was the outcome of this study. Standardized equipment and procedures were used for measuring hemoglobin and MUAC values. Hemoglobin concentration was measured by taking a finger-prick blood sample of each pregnant woman using a HemoCue Hb 201 (HemoCue AB, Angelholm, Sweden).After the site was cleaned with disinfectant, a prick was made on the tip of the middle finger. The first drop of blood was cleaned off, and the second drop was collected to fill the microcuvette, which was then placed in the cuvette holder of the HemoCue Hb analyzer for measuring hemoglobin concentration (32). Pregnant women are considered anemic if their hemoglobin level is below 11 g/dL in the first and third trimesters and 10.5 g/dL in the second trimester of pregnancy. The severity of anemia during pregnancy is classified into three: mild anemia (10–10.9 g/dL), moderate anemia (7.0–9.9 g/dL), and severe anemia (<7.0 g/dL). As per WHO recommendation, hemoglobin values were adjusted for every 500 m for altitudes exceeding 1,000 m above sea level (11). The altitude of the study area ranges from 1,735 to 2,917 m above sea level (33). As a result, 0.5–1.3 g/dL were deducted from the individual hemoglobin measurement values. MUAC of the left arm was measured using an adult MUAC non-stretchable measuring tape with no clothing on the arm. The women were asked to sit and bend their left arm at 90°. The measuring tape was placed at the tip of the shoulder (acromion) and the tip of the elbow (olecranon process). The midpoint of the tips was marked, and its circumference was measured to the nearest 0.1 cm. This measurement was repeated three times, and the mean of three measurements was taken. Pregnant women with MUAC measurements below 23 cm were classified as malnourished (34).

#### 2.5.2. Independent variables

Indicators used to measure women's empowerment for this study were based on previous work (20–22, 35). This suggested that women can be empowered at individual and household levels; agency and resources as pillars of women's empowerment are reflected in familial/interpersonal, economic, sociocultural, time, and psychological dimensions. Twenty-nine items were included in the survey to capture women's empowerment. Questions relating to attitude, decision-making, and self-security powers were used to measure agency, whereas resource was measured using income- and time-related indicators. All the variables were recoded to make them suitable for analysis and interpretation. Scores to the indicators were given, assuring higher values indicate a higher empowerment level (27, 36–38).

#### 2.5.3. Control variables

Pregnant women's characteristics (educational level, age, occupation, place of residence, and access to mass media), partner's educational and occupation statuses, household characteristics (household wealth index), and obstetric factors such as parity (number of children), gestational age, and pregnancy gap were the potential control variables. Age, gestational age, parity, and pregnancy gap were continuous variables; the rest were categorical. The pregnant women's and partners' educational levels were categorized into illiterate, primary, secondary, diploma, first degree, second degree, and above. The occupational status of the women and partners were grouped into government employee, private employee, farmer, merchant, daily laborer, and student; housewife was an additional option for women. Place of residence was dichotomized: if the respondent lived in an urban or rural area. The pregnant women were also asked whether they had access to either electronic media or written material or if nothing was used to assess their access to mass media.

Data on household assets were collected based on DHS questions. The 12 questions considered for wealth index calculation were recoded into binary variables i.e., 0 and 1. Questions with “yes” and “no” options and with more than two options were transformed into bivariate variables. Drinking, cooking, and washing water source, toilet facility, and roof and floor materials were categorized into improved and unimproved, while cooking fuel was considered solid and clean based on DHS classification (39). Then, principal component analysis was conducted to calculate the household wealth index. Households were categorized into high, medium, and low quartiles based on their household wealth index score.

### 2.6. Statistical analyses

Data were entered using EpiData 3.1. Data cleaning and analyses were done using STATA version 16.0 (Stata Corporation, College Station, TX). A descriptive analysis was conducted to examine the background characteristics of the study participants. Exploratory factor analysis (EFA) was conducted to identify the structure of individual items and dimensions suitable for measuring women's empowerment (40). Then, it was cross-validated using confirmatory factor analysis (CFA). A correlation test was first conducted on the 29 items, which measured empowerment to see their strength for factor analysis. The data were divided into two groups using a random uniform distribution to conduct EFA with one data sample and CFA with the second data sample. Running the factor test showed that the *x*^2^ for the Bartlett test of sphericity was significant at *p* < 0.001, and the Kaiser–Meyer–Olkin test result was 0.782, which implied that the strength of variables correlation was sufficient for factor analysis. Then, the polychoric correlation was calculated, which is recommended for categorical variables (41). EFA was conducted on the polychoric correlation matrix as input rather than the raw variables. Oblique rotation was used to rotate the indicators. Individual indicators with a rotated factor loading >0.5 (42) were used to construct the different dimensions of women's empowerment. Indicators with significant negative pattern coefficients, single loaders to a factor, and cross loaders to more than one factor were removed. Moreover, indicators that contributed to a lower reliability coefficient for a factor (Cronbach's alpha <0.7) were removed one at a time. A factor with poor reliability was removed at the end. As one indicator was removed, analysis was done on the remaining indicators. Finally, six factors were obtained based on eigenvalues >1 and the interpretability of factors (42, 43). The number of indicators was reduced to 22. Cronbach's alpha coefficients for the six factors were between 0.74 and 0.84.

CFA was conducted on the remaining randomly split half data sample to validate the fitness of the factors identified from the EFA. Model fitness was determined based on acceptable cutoff values of the root-mean-square error of approximation (RMSEA) < 0.08, Bentler comparative fit index (CFI) ≥ 0.90, Tucker–Lewis index (TLI) ≥ 0.90, and standardized root mean square residual (SRMR) < 0.08 (44, 45). To improve the initial model fitness, four indicators with *R*-squared values less than 0.2 were removed since they were sources of very high error levels (44). When correlation occurred in the error terms of individual indicators measuring the same factor, the one with the lower factor loading indicator was removed. Hence, one indicator was removed. Finally, the RMSEA, CFI, TLI, and SRMR values were 0.063, 0.928, 0.901, and 0.05, respectively, implying that the model fit to the data was acceptable. The CFA confirmed the six factors, and 17 indicators were retained with pattern coefficients ranging from 0.54 to 0.94. The total variance explained by the dimensions was 83.5%.

The six factors/dimensions, which operationalized women's empowerment, were identified as “household decision-making power, economic, communication, psychological, assertiveness and time.” Details of the indicators that measured the six dimensions are found in Table 1. The *decision-making* dimension refers to the participation of women in household matter decisions. A score of 1 was given if the woman participated in the decision-making either alone or jointly with her husband/partner, and a score of 0 was assigned if decision was made by her partner or someone else. The scoring of answers for *economic dimension* was regarding women's engagement in cash income activities, having savings of their own, and access to household money (1 = yes; 0 = no). A woman was given a score of 1 if she made decisions alone or jointly, while 0 score was given if others made decisions regarding her earnings. The *communication* dimension deals with discussions on feelings and household matters between partners. Scores of 2, 1, and 0 were given for the indicators' responses of “always,” “sometimes,” and “never,” respectively. The *Psychological* dimension addresses issues related to the women's self-esteem and self-confidence. Response of “always” was assigned 1, while “not at all” and “sometimes” were assigned 0 for the indicators that examined this dimension. *The assertiveness* dimension assessed the certainty level of women in defending their rights. The women who answered “completely sure” were given a score of 2, whereas “somewhat sure” got a score of 1. Those who were “neither sure nor unsure,” “somewhat unsure,” and “not at all sure” were given a score of 0. To assess the *time* dimension*,* values of 0, 1, and 2 were assigned if the indicators' response to the average time spent on housework daily and the actual time spent on housework the previous day was greater than 10 h, between 5 and 10 h, and less than 5 h, respectively.

**Table 1:** Level of pregnant women’s empowerment in West Shewa Zone, Central West Ethiopia, 2021(*n* = 1,453).^a^

Dimensions	Indicators	Mean (SD)	*n* (%)
Household decision-making power	Decision on what food to buy and consume		1,248 (85.9)
	Decision on food prepared every day		1,323 (91.1)
	Decision on own healthcare		1,318 (90.7)
	Decision on daily needs household purchases		1,271 (87.5)
	Mean score of decision-making power (out of 0–4 total points)	3.6 (1.1)	
	% women > mean		1,149 (79.1)
Economic	Engagement with cash income activities		978 (67.3)
	Cash savings of own		498 (34.3)
	Decision on own earning use		1,049 (72.2)
	Direct access to household money for use		1,099 (75.6)
	Mean score of economic dimension (out of 0–4 total points)	2.5 (1.2)	
	% women > mean		866 (59.6)
Communication	Discussion between partners about worries or feelings		359 (24.7)
	Discussion between partners on household money spending		485 (33.4)
	Mean score of communication (out of 0–4 total points)	2.4 (1.1)	
	% women > mean		564 (38.8)
Psychological	Self-confidence in problem solving		574 (39.5)
	Feeling like an important household member		958 (65.9)
	Feeling household members respect your opinion		725 (49.9)
	Mean score of psychology	1.6 (1.2)	
	% women > mean (out of 0–3 total points)		772 (53.1)
Assertiveness	Sure of going to a health facility in the presence of partner’s objection		372 (25.6)
	Sure of using family planning, even if the partner objects		444 (30.6)
	Mean score of assertiveness	1.7 (1.5)	
	% women > mean (out of 0–4 total points)		741 (51.0)
Time	Daily time expenditure on housework		664 (45.7)
	Time spent on housework the previous day		790 (54.4)
	Mean score of time	3.0 (1.0)	
	% women > mean (out of 0–4 total points)		854 (58.8)
Total women’s empowerment mean score (out of 2–23 total points)		14.7 (4.1)	
% women > mean			788 (54.2)

^a^ Descriptive analysis was conducted on women’s empowerment indicators and dimensions obtained from factor analysis. Categorical variables were expressed as frequencies and percentages, while used for continuous variables were expressed as means (standard deviations).

SD, standard deviation.

The six dimensions were used to calculate the composite women's empowerment. Higher values of the indicator questions under each dimension represented high empowerment, whereas lower values denoted low empowerment. A cutoff point was applied for each dimension and composite dimensions to categorize the women into two, empowered and not empowered, based on the proportion of women with total scores above the population mean vs. those with scores at or below the population mean. Each dimension and indictor was given equal weight during the calculation of women's empowerment status.

First, the socioeconomic and demographic characteristics of the women and households and obstetric-related variables significantly associated with the women's nutritional status were identified in bivariate analysis and included in a model as control variables. Then, multiple logistic regression analysis was conducted. Control variables significant at *p* < 0.2 were included in multiple logistic regression analysis. Two models were used. In the first model, the composite women's empowerment score was used to assess its association with outcome variables. The associations of the individual dimensions of women's empowerment with pregnant women's nutritional status were investigated in the second model. The variation inflation factor of the independent variables in the models was below 10. The likelihood-ratio chi-square and *R*² tests were used to assess model fit. *p* < 0.05% and 95% confidence intervals were used to declare a significant association of results.

## 3. Results

### 3.1. Descriptive characteristics of study participants

Out of 1,453 pregnant women, the majority (61.2%) resided in urban areas. Their mean age was 28.1 (SD 5.3) years, with a range of 18–44 years. The mean number of children the pregnant women had was 2.4 (SD 1.5), with a minimum of 1 and a maximum of 8. The mean birth interval between the current and previous pregnancies was 2.9 (SD 1.7) years, with a range of 1–15 years (Table 2).

**Table 2:** Descriptive statistics of sociodemographic and obstetric characteristics of pregnant women in West Shewa Zone, Central West Ethiopia, 2021 (*n *= 1,453).

Characteristics	Frequency	%
Sociodemographic characteristics		
Age in years		
<19	83	5.7
20–29	797	54.9
>30	573	39.4
Residence		
Rural	564	38.8
Urban	889	61.2
Women's education level		
Illiterate	310	21.3
Elementary	343	23.6
Primary	160	11.0
Secondary	272	18.7
Diploma	218	15.0
First degree and above	150	10.3
Women's occupation		
Government employee	300	20.7
Private employee	164	11.3
Trading/merchant	171	11.8
Daily laborer	60	4.1
Farmer	306	21.1
Housewife	421	29.0
Student	31	2.1
Household wealth index		
Low	489	33.7
Middle	495	34.1
High	469	32.3
Obstetric characteristics		
First pregnancy		
Yes	606	41.7
No	847	58.3
Gestational age		
2nd trimester	108	7.4
3rd trimester	1,345	92.6
Number of living children		
<2	516	60.9
3–4	243	28.7
>5	88	10.4
Birth interval between the current and previous pregnancies in years		
<3	396	46.8
>3	451	53.3

### 3.2. Nutritional status of pregnant women

Of 1,453 pregnant women, 18.5% (269) (95% CI: 16.60, 20.60) were anemic (hemoglobin < 11 g/dL) without altitude adjustment. After altitude adjustment, the prevalence increased to 32.1% (466) (95% CI: 29.72, 34.52). Of the 466 anemic pregnant women, 10 (2.2%) had severe, 250 (53.6%) had moderate, and 206 (44.2%) had mild anemia. About 34 (7.3%) and 432 (92.7%) of them were in the second and third trimesters, respectively. The mean hemoglobin value was 12.4 g/dL (SD 1.7). Of 1,453 pregnant women, 306 (21.1%) (95% CI: 19.04, 23.24) were malnourished (MUAC <23 cm). The mean MUAC value was 25.0 cm (SD 2.2).

### 3.3. Women's empowerment status

Of 1,453 pregnant women, 788 (54.2%) (95% CI:51.66, 56.79) were empowered overall. The highest and least empowerment levels of pregnant women were identified in household decision-making power and communication dimensions respectively. Most of the pregnant women were empowered to make decisions regarding food prepared for the household. However, of 1,453 pregnant women, only 498 (34.3%) had cash savings of their own. Less than half of them had frequent communication with their partners. Similarly, the number of pregnant women who were assertive enough to accomplish their desires even in the presence of obstacles was very low (<31%). Around half of them had a good sense of self and self-confidence. Of 1,453 pregnant women, about 756 (54.3%) spent more than 6 hours on housework daily, whereas around 33 (3%) spent more than 10 hours on housework daily. Table 1 summarizes the results of the empowerment level of pregnant women. The percentage in the table is for indicators' responses with the highest score per recording.

### 3.4. Associations between women's empowerment and pregnant women's nutritional status

In bivariate analysis, except age, gestational age, and pregnancy gap, all the rest control variables (residence place, wealth index, women's and their partners' educational level and occupational status, access to mass media, first pregnancy, and parity) were significantly associated with the anemia status of the pregnant women (*p* < 0.05). Age, residence place, wealth index, women's educational level and occupational status, partners' occupational status, and first pregnancy had a significant association with the MUAC level of the pregnant women, while educational status of partner, mass media exposure, pregnancy gap, gestational age, and parity had *p*-values above 0.2.

The association results of composite and individual dimensions of women's empowerment with pregnant women's nutritional outcomes are presented in Table 3. Pregnant women's composite empowerment score was significantly and positively associated with the anemia status and MUAC level. Empowered pregnant women were more likely not to be anemic (AOR = 1.9, 95% CI: 1.47, 2.43) and have MUAC ≥ 23 cm (AOR = 1.8, 95% CI: 1.32, 2.35) than not-empowered women. On the other hand, only economic and assertiveness dimensions were significantly associated with pregnant women's anemia status. Economically empowered pregnant women had 1.7 higher odds of being free from anemia than those not empowered (AOR = 1.7, 95% CI: 1.26, 2.22). Pregnant women who were empowered in the assertiveness dimension were 1.9 times more likely to be anemia-free (AOR = 1.9, 95% CI: 1.46, 2.38) than women who were not empowered in the assertiveness dimension. Similarly, only two dimensions were positively and significantly associated with the MUAC level of pregnant women. Pregnant women empowered in household decision-making power had higher odds of having MUAC ≥ 23 cm (AOR = 1.6, 95% CI: 1.19, 2.22) than those not empowered in this dimension. Pregnant women empowered in the psychological dimension were 1.4 times more likely to have MUAC ≥ 23 cm (AOR = 1.4, 95% CI: 1.04, 1.85) than those not women in the psychological dimension.

**Table 3:** Associations of women’ empowerment with pregnant women's anemia status and MUAC level in the West Shewa Zone, Ethiopia. (*n* = 1,453).^a^

	Anemia status^b^				MUAC^c^			
Model	Anemic	Not anemic	AOR (95% CI for AOR)	P	MUAC <23 cm	MUAC ≥ 23 cm	AOR (95% CI for AOR)	*p*
Model I								
Composite women's empowerment								
Not empowered	275 (18.9)	390 (26.8)	Ref		183 (12.6)	482 (33.2)	Ref	
Empowered	191 (13.2)	597 (41.1)	1.9 (1.47, 2.43)	0.000	123 (8.5)	665 (45.8)	1.8 (1.32, 2.35)	0.000
Model statistics								
LR (Chi-square)	89.2				110.4			
*R*²	0.049				0.074			
*p*	0.000				0.000			
Model II								
Decision-making power								
Not empowered	120 (8.3)	184 (12.7)	Ref		95 (6.5)	209 (14.4)	Ref	
Empowered	346 (23.8)	803 (55.3)	1.2 (0.92, 1.63)	0.164	211 (14.5)	938 (64.6)	1.6 (1.19, 2.22)	0.003
Economic								
Not empowered	246 (16.9)	341 (23.5)	Ref		159 (10.9)	428 (29.5)	Ref	
Empowered	220 (15.1)	646 (44.5)	1.7 (1.26, 2.22)	0.000	147 (10.1)	719 (49.5)	1.3 (0.91, 1.73)	0.172
Communication								
Not empowered	314 (21.6)	575 (39.6)	Ref		200 (13.8)	689 (47.4)	Ref	
Empowered	152 (10.5)	412 (28.4)	1.1 (.87, 1.50)	0.340	106 (7.3)	458 (31.5)	1.1 (0.77, 1.43)	0.764
Psychological								
Not empowered	246 (16.9)	435 (29.9)	Ref		169 (11.6)	512 (35.2)	Ref	
Empowered	220 (15.1)	552 (38.0)	1.2 (0.92, 1.53)	0.186	137 (9.4)	635 (43.7)	1.4 (1.04, 1.85)	0.027
Assertiveness								
Not empowered	281 (19.3)	431 (29.7)	Ref		165 (11.4)	547 (37.7)	Ref	
Empowered	185 (12.7)	556 (38.3)	1.9 (1.46, 2.38)	0.000	141 (9.7)	600 (41.3)	1.1 (0.80, 1.39)	0.702
Time								
Not empowered	236 (16.2)	363 (25.0)	Ref		151 (10.4)	448 (30.8)	Ref	
Empowered	230 (15.8)	624 (43.0)	1.2 (0.89, 1.54)	0.273	155 (10.7)	699 (48.1)	1.3 (0.98, 1.82)	0.070
Model statistics								
LR (chi-square)	124.1				120.7			
*R*²	0.068				0.081			
*p*	0.000				0.000			

^a^ Multiple logistic regression analyses were done to find the associations of individual and composite women's empowerment dimensions with anemia and MUAC status during pregnancy.

^b^ Adjusted for age, residence place, wealth index, women's and their partners’ educational level and occupational status, access to mass media, and first pregnancy.

^c^ Adjusted for age, residence place, wealth index, women's educational level, occupational status of women and their partners, and first pregnancy.

AOR, adjusted odds ratio; CI, confidence interval.

## 4. Discussion

This study used multiple dimensions of pregnant women's empowerment to examine its relationship with their nutrition outcomes in West Shewa Zone in Ethiopia. It used the resource–agency–achievement framework to assess pregnant women's nutrition outcomes. The contributions of the composite and the six individual dimensions of women's empowerment, including household decision-making power, economic, communication, psychological, assertiveness, and time, were analyzed.

Key findings of this study pointed out that poor nutritional status of pregnant women was common in the study area. Moreover, the overall empowerment of pregnant women was significantly and positively associated with pregnant women's nutritional status. Household decision-making power, economic, psychological, and assertiveness dimensions of pregnant women's empowerment showed a positive association with one of the nutrition outcomes, while communication and time dimensions were not significantly associated with any of the nutrition outcomes.

The current study revealed that 32.1% of pregnant women were anemic. Similar results were reported by studies conducted in different regions of Ethiopia. For instance, 33.1% of pregnant women in Eastern Ethiopia (8) and 32.8% of pregnant women in Arba Minch (46) were found to be anemic after altitude adjustment. However, lower prevalence has been reported by studies in Western Ethiopia (17.8%) (47) and Southeast Ethiopia (27.9%) (48). Not considering altitude adjustment might be among the reasons for differences. Anemia is a moderate public health problem in the study area according to the WHO classification of the public health significance of anemia (11). In this study, 21.1% of the pregnant women were malnourished (MUAC < 23 cm). Other studies in Ethiopia have also reported a significant proportion of pregnant women as malnourished (7, 9, 18). This implies the necessity to develop effective interventions and nutritional status improvements among pregnant women.

Significant and positive associations were found between composite pregnant women's empowerment, anemia status, and MUAC level. Overall empowered pregnant women had fewer odds of being anemic or malnourished than not-empowered pregnant women. The results suggest that increasing the overall empowerment level of pregnant women is crucial for reducing malnutrition among them. As women's empowerment levels increase, they have greater knowledge of nutrition and health and greater decision-making power, income control, and time to exercise that knowledge in their self-care (49). Supporting findings have been reported in other studies investigating the associations between non-pregnant women's empowerment and their nutritional status even if different women's empowerment indicators were utilized. For instance, in India, women's empowerment in the nutrition index was significantly associated with women's anemia status (50). Another Indian study that used women's empowerment in the agriculture index for measuring women's empowerment has pointed out a significant positive association between women's empowerment in the agriculture score and the anemia status of women (51). Women's hemoglobin level was positively associated with their instrumental agency (influence in household decision-making) among East African women (52).

Only economic and assertiveness dimensions were significantly associated with anemia status, whereas the two dimensions significantly associated with the MUAC level of pregnant women were decision-making power and psychological dimensions, suggesting that the different dimensions of pregnant women's empowerment have varying influences on the nutritional status of pregnant women.

Association results between decision-making power and maternal nutrition outcomes have been inconsistent. Empowerment in household decision-making was associated with better maternal nutritional status (27, 28, 53), while null associations were also reported (54). In this study, pregnant women who were empowered in the household decision-making dimension were less likely to be malnourished than those who were not empowered. Empowerment in women's decision-making can ensure improved nutritional status by allowing to make decisions regarding purchases, preparation, and consumption of food items for the household. However, the absence of any association between household decision-making power and anemia status was unexpected. This can be owing to the multifactorial causes of anemia, including micronutrient deficiencies, parasitic and chronic infections, geographical location, and dietary practice. Moreover, women in Ethiopia are responsible for most of the household chores, including the management of food-related issues, as evidenced in this study. Therefore, better decision-making power of the women can lead to better nutritional status at the household level in their family and may not be directly observed in the nutritional status of the pregnant women. The result of this study also implies that pregnant women's decision-making power may also be influenced by other dimensions of women's empowerment, for instance, economic empowerment. Having higher household decision-making power may not lead to improved nutritional status among pregnant women if there is no economic empowerment. Women's economic empowerment may enable women to afford quality food; it can strengthen their household decision-making power, which contributes to improving their wellbeing (55). Interventions targeting these women would benefit by promoting empowerment in multiple dimensions and encouraging them to transfer their decision-making power to self-care as well. Yet, a similar result was found in the analysis of the 2016 Ethiopia Demographic and Health Surveys data, which indicated that women's decision-making does not influence their anemia status (56). On the contrary, a study in five East African countries found that women's household decision-making is positively associated with their blood hemoglobin level (52). Differences in indicators used for decision-making measurements, study settings, and study participants may contribute to varying results. In the current study, only pregnant women were included instead of non-pregnant women in the referred studies (52, 56).

Economically empowered pregnant women were less likely to be anemic in this study. A related study on non-pregnant women's empowerment and their nutritional status indicated that control over income was linked with the better nutritional status of women (53). For instance, the greater magnitude of anemia has been linked to the low decision-making capacity of women to access resources and health information (56). Participating in income-generating activities can improve access and control over resources. This enhances their decision-making power and control over their and family's life. Income improves self-confidence and self-esteem (57). Women's participation in household decision-making and their ability to purchase food positively impact the availability of diverse and nutrient-rich diets in the household and, consequently, adequate dietary diversity intake among women (58, 59), reducing the likelihood of being anemic. On the other hand, the MUAC status of the pregnant women was not related to their economic empowerment status. Nutritional status during pregnancy depends not only on adequate dietary intake but also on sociocultural factors, energy expenditure, and nutrition knowledge (60, 61). For instance, women work longer hours when both paid and unpaid work is considered (62). Women in Ethiopia are involved in unpaid and tiresome household work, making them the most disadvantaged members of society (63). This circumstance may increase the probability of energy expenditure, resulting in low MUAC. Women may also face time poverty for self-care due to heavy workloads. Unemployed and farmer women in Ethiopia were particularly identified as the most malnourished (28).

The significant and positive association of psychological empowerment with MUAC status in this study shows the important role pregnant women's self-confidence and esteem have on nutrition outcomes during pregnancy. Other related studies also found that women's self-confidence was related to their and their children's nutritional status (27, 64, 65). Psychological empowerment is characterized by a sense of perceived control, competence, and goal internalization (66). Hence, it promotes the chance of engaging in healthy behaviors including healthy food choices and healthy dietary practices, thereby contributing to better nutritional status. They have reported increased self-confidence to positively influence nutrition outcomes. On the other hand, the insignificant association between the psychological dimension and the anemia status of pregnant women in this study implies the need for further investigation. The result may reflect the presence of other relevant factors in the middle concealing the relationship between pregnant women's psychological empowerment and anemia status during pregnancy.

This study shows that assertive pregnant women were less likely to be anemic than non-assertive pregnant women. Assertive women are likely to stand up for their rights, say what they want or believe in, and object to unreasonable requests (67). This can be reflected in their access to resources and health service utilization, which in turn can be manifested in their nutritional status. It is evidenced that assertive women are confident and self-assured. They are more likely to be empowered psychologically. Hence, the assertiveness dimension was expected to associate with the MUAC level of pregnant women, in line with the psychological dimension. Nevertheless, empowerment in the assertiveness dimension was found not to relate to MUAC status. The opposing association results of the specific dimensions with the outcome variables suggest the unique influences of the dimensions on nutrition outcomes. Assertiveness is important for other women's empowerment dimensions to be effective (68). However, assertiveness alone may not ensure better nutritional status, as evidenced in this study. Self-confidence may enhance the capability of using the available resources and circumstances, but assertiveness alone may not bring the expected outcome without such conditions. Even if the pregnant women were assertive, they might not be empowered in the other empowerment dimensions. This may be the reason for the null association observed in this study.

Communication and time dimensions were not associated with pregnant women's nutrition outcomes in this study. On the contrary, related studies pointed out that, generally, high women's workload was linked with poor nutritional status of the women (53, 69). Women may work more to increase the quantity and quality of food available to their households, but longer work hours may also increase their energy expenditure, resulting in poor nutrition outcomes (69). It was also noted that intrahousehold communication may help achieve improved maternal nutrition outcomes (36). Communication helps women to express and share needs and thoughts. Empowered communication helps women to enhance their decision-making power, economically and politically (55, 70). The unexpected results in this study may imply that the better nutritional status of pregnant women is not necessarily related to empowerment in all dimensions of women's empowerment. In line with the above explanations, the interconnectedness of the empowerment dimensions may be one of the reasons for the insignificant association results. Empowerment in a specific dimension that may not necessarily guarantee better nutritional status among pregnant women; rather, it is the cumulative empowerment of the different dimensions. This is reflected in this study considering significant relations between overall empowerment and nutrition outcomes of pregnant women. Moreover, it has been underlined that the nutritional status of pregnant women depends on multiple factors such as dietary habit, access to health services, infectious diseases, and knowledge (71) besides the independent and control variables considered in this study. Excluding these factors may have affected our study results. In general, the contextual nature of the different associations between women's empowerment dimensions and pregnant women's nutrition outcomes may be understood in-depth by qualitative research. Moreover, qualitative investigations may help identify crucial and unmeasured women's empowerment features that are related to pregnant women's nutrition outcomes and determine the appropriateness of the indicators used for capturing women's empowerment in this study. Several indicators were dropped during factor analysis. This may have affected our results.

Empowering women has been the central aim of the National Women's Policy of Ethiopia through enhancing women's participation and benefit from the economic, social, and political activities by creating an enabling environment for women (72). Ethiopia has integrated sustainable development goals (SDGs), including SDG 5 on women's empowerment, in its national development plan from 2019/2020 to 2029/2030. The national development plan aims at improving maternal health, and empowering women is identified as one of the strategies for its achievement (73). Moreover, improving maternal health and nutrition is among the targets of the Ministry of Health of Ethiopia, as they are indicated in its Health Sector Transformation Plan II 2020/2021–2024/2025 (74). The plan is prepared to be implanted in all regions of Ethiopia. Therefore, the ministry's effort to improve pregnant women's nutrition in the study area could benefit from this study's findings by considering the significant women's empowerment components related to nutrition for implementation.

This study has limitations. Cross-sectional data were used, so causality could not be inferred. Any causal inference is tentative. Reciprocal effects may exist. Only partnered pregnant women attending antenatal care units in public health facilities were included in this study. This limits the generalizability of the findings to the excluded segments of women. The study tried to use multiple dimensions to assess women's empowerment, and indicators for women's empowerment measurement in this study were developed based on existing literature. However, the sociocultural factors of the study area were not considered. Hence, there may be unaddressed aspects of women's empowerment. There are limited studies that assessed the link between pregnant women's empowerment and their nutrition outcomes. As a result, most of the comparisons in this study were with non-pregnant women.

The findings of this study suggest that overall empowerment of pregnant women was positively associated with anemia status and MUAC level during pregnancy. Policies and programs that aim to reduce poor nutrition outcomes among pregnant women in the study area may benefit from interventions that promote, particularly, decision-making power, economic, psychological, and assertiveness dimensions of pregnant women.

## Data Availability

The original contributions presented in the study are included in the article/**Supplementary Materials**, further inquiries can be directed to the corresponding author.
