# Supplementary material for: Predicting nutritional status during pregnancy by women's empowerment in West Shewa Zone, Ethiopia
Source: Front Glob Womens Health. 2023 Jun 19;4:1147192. doi: 10.3389/fgwh.2023.1147192 (PMC10316787; doi:10.3389/fgwh.2023.1147192)
Supplement: Supplementary file 1 [file Table1.docx]

Supplementary Table 1. Questionnaires on socio-demographic, obstetrics characteristics and women’s empowerment.

| Part I. Socioeconomic and demographic characteristics of respondents | | | | | | | | | | | |
| --- | --- | --- | --- | --- | --- | --- | --- | --- | --- | --- | --- |
| S. No. | | | Questions | Response categories | | | | | | | Skip |
| 101 | | | District of the respondent | ____________ | | | | | | |  |
| 102 | | | Kebele of the respondent | ____________ | | | | | | |  |
| 103 | | | Residence place of the respondent | 1. Urban 2. Rural | | | | | | |  |
| 104 | | | How old are you? (in years) | _________________ years | | | | | | |  |
| 105 | | | What is your educational level completed? | 1. Illiterate 2. Read and write but with no formal education 3. Elementary/ Grade 1-6 4. Primary school/ Grade 7-8 5. Secondary school/ Grade 9-12 6. Diploma 7. Degree 8. Masters 9. Above masters | | | | | | |  |
| 106 | | | What is the educational level of your partner? | 1. Illiterate 2. Read and write but with no formal education 3. Elementary/ Grade 1-6 4. Primary school/ Grade 7-8 5. Secondary school/ Grade 9-12 6. Diploma 7. Degree 8. Masters 9. Above masters | | | | | | |  |
| 107 | | | What is your occupation? | 1. Government employee 2. Private employee 3. Trading/merchant 4. Daily laborer 5. Farmer 6. Housewife 7. Student 8. Other (specify) _____ | | | | | | |  |
| 108 | | | What is your husband/partner’s occupation? | 1. Government employee 2. Private employee 3. Trading/merchant 4. Daily laborer 5. Farmer 6. Student 7. Other (specify) _____ | | | | | | |  |
| 109 | | | For which mass media do you have access? (check for all responses) | 1. Radio 2. Television 3. Newspaper 4. Others specify_______ | | | | | | |  |
| 110 | | | What is the main source of drinking water for members of your household? | 1. Piped water into dwelling 2. Piped water to neighbor 3. Public tap/standpipe 4. Protected well 5. Unprotected well 6. Protected spring 7. Unprotected spring 8. Rainwater 9. Surface water (river/dam/lake/pond/stream/canal/   irrigation channel)   1. Other (specify)_____________ | | | | | | |  |
| 111 | | | What is the main source of water used by your household for other purposes such as cooking and hand washing? | 1. Piped water into dwelling 2. Piped water to neighbor 3. Public tap/standpipe . 4. Protected well 5. Unprotected well 6. Protected spring 7. Unprotected spring 8. Rainwater 9. Surface water (river/dam/lake/pond/stream/canal/   irrigation channel)   1. Other (specify)___________ | | | | | | |  |
| 112 | | | How is your residential house ownership? | 1. Own home 2. Rented home 3. Other (specify)_______ | | | | | | |  |
| 113 | | | Do you have a separate room which is used as a kitchen? | 1. Yes 2. No | | | | | | |  |
| 114 | | | What kind of toilet facility do members of your household usually use? | 1. Traditional pit latrine 2. Ventilated improved pit latrine 3. Flush or pour flush toilet 4. Open field 5. Other (specify)_______ | | | | | | |  |
| 115 | | | Does your household have radio? | 1.Yes  2. No | | | | | | |  |
| 116 | | | Does your household have television? | 1.Yes  2. No | | | | | | |  |
| 117 | | | Does your household have refrigerator? | 1.Yes  2. No | | | | | | |  |
| 118 | | | Does your household have an electric mitad? | 1.Yes  2. No | | | | | | |  |
| 119 | | | What type of fuel does your household mainly use for  cooking? | 1. Electricity 2. Biogas 3. Kerosene 4. Charcoal 5. Wood 6. Straw/shrubs/grass 7. Agricultural crop 8. Animal dung 9. Other (specify)_______ | | | | | | |  |
| 120 | | | What is the main material of the roof of your dwelling? | 1. Thatched 2. Corrugated iron sheet 3. Plastic sheet 4. Other (specify)_______ | | | | | | |  |
| 121 | | | What is the main material of the floor of your dwelling? | 1. Earth/sand 2. Dung 3. Rudimentary floor with   wood/bamboo   1. Finished floor with cement 2. Other (specify)_______ | | | | | | |  |
| Part II. Obstetrics related questions | | | | | | | | | | | |
| 201 | | What is the gestational age of the pregnancy? | | | | ____(weeks) | | | |  | |
| 202 | | Is this your first pregnancy? | | | | 1. Yes 2. No | | | | If the answer is yes, go to question 301 | |
| 203 | | How many child/children do you have? | | | | __________ | | | |  | |
| 204 | | If the answer for question 203 is above 1, what is the age difference between the current pregnancy and previous pregnancy? | | | | _________year(s) | | | |  | |
| Part III. Women’s empowerment related questions | | | | | | | | | | | |
| Familial/interpersonal dimension | | | | | | | | | | | |
| 301 | Who usually makes decisions on what food to buy and consume? | | | | 1. You 2. Your husband 3. You and your husband together 4. Mother‐ or father‐in‐law 5. Someone else(specify)______ | | | | | |  |
| 302 | Who usually makes decisions about what food is prepared every day? | | | | 1. You 2. Your husband 3. You and your husband together 4. Mother‐ or father‐in‐law 5. Someone else(specify)______ | | | | | |  |
| 303 | Who usually makes decisions about health care for yourself? | | | | 1. You 2. Your husband 3. You and your husband together 4. Mother‐ or father‐in‐law 5. Someone else(specify)______ | | | | | |  |
| 304 | Which member of your household usually makes decisions about making household purchases for daily needs (for example: onion, oil,…)? | | | | 1. You 2. Your husband 3. You and your husband together 4. Mother‐ or father‐in‐law 5. Someone else(specify)______ | | | | | |  |
| 305 | Which member of your household usually makes decisions about the number of children to have? | | | | 1. You 2. Your husband 3. You and your husband together 4. Mother‐ or father‐in‐law 5. Someone else(specify)______ | | | | | |  |
| 306 | How often do you and your husband discuss your worries or feelings? | | | | 1. Never 2. Seldom 3. Sometimes 4. Often 5. Always | | | | | |  |
| 307 | How often do you and your husband discuss what to spend household money on? | | | | 1. Never 2. Seldom 3. Sometimes 4. Often 5. Always | | | | | |  |
| 308 | Do you get help from your husband with the household duties? | | | | 1. Always  2. Sometimes  3. Not at all | | | | | |  |
| Economic dimension | | | | | | | | | | | |
| 309 | Are you engaged in any activities (jobs) with cash income? | | | | | | 1. Yes  2. No | | | |  |
| 310 | Do you have any cash savings of your own? | | | | | | 1. Yes  2. No | | | |  |
| 311 | Do you own any assets (for example, land, house, cattle or shop) that could help you generate income? | | | | | | 1. Yes  2. No | | | |  |
| 312 | Who usually decides how your husband’s/partner’s earnings will be used? | | | | | | 1. You 2. Your husband 3. You and your husband together 4. Mother‐ or father‐in‐law 5. Someone else(specify)______ | | | |  |
| 313 | Who usually decides how the money you earn will be used? | | | | | | 1. You 2. Your husband 3. You and your husband together 4. Mother‐ or father‐in‐law 5. Someone else(specify)______ | | | |  |
| 314 | Do you have direct access to household money in your hand to use? | | | | | | 1. Yes  2. No | | | |  |
| 315 | Is there amount of money you can spend freely? | | | | | | 1. Yes  2. No | | | |  |
| Socio-cultural dimension | | | | | | | | | | | |
| 316 | Do you think pregnant women should eat more during pregnancy than other times? | | | | | | | 1. Strongly disagree  2. Disagree  3. Neither agree nor disagree  4. Agree  5. Strongly agree | | |  |
| 317 | Do you think pregnancy complications (such as preeclampsia ) are related with dietary practice of the pregnant mother? | | | | | | | 1. Strongly disagree  2. Disagree  3. Neither agree nor disagree  4. Agree  5. Strongly agree | | |  |
| 318 | Do you think birth outcomes (such as birth weight, preterm birth, stillbirth, postpartum hemorrhage) are related with dietary practice of the pregnant mother? | | | | | | | 1. Strongly disagree  2. Disagree  3. Neither agree nor disagree  4. Agree  5. Strongly agree | | |  |
| 319 | Do you usually meet with other women in your community to discuss and receive information on health and/or nutrition related issues? | | | | | | | 1. Yes  2. No | | |  |
| Time dimension | | | | | | | | | | | |
| 320 | For how many hours do you work daily? | | | | | | | | ______hours | |  |
| 321 | How long do you usually spend on housework daily? | | | | | | | | ______hours | |  |
| 322 | How long did you spend on housework the previous 24 hours? | | | | | | | | ______ hours | |  |
| 323 | How would you rate your satisfaction with your time available for leisure activities such as visiting neighbors, time for your own, watching TV, listening to the radio, resting? | | | | | | | | 1. Very unsatisfied  2. Less unsatisfied  3. Satisfied  4. Less satisfied  5. Very satisfied | |  |
| 324 | Do you have time to participate in community activities in your community? | | | | | | | | 1. Yes  2. No | |  |
| Psychological dimension | | | | | | | | | | | |
| 325 | How sure are you that you could use family planning, even if your husband did not want to? | | | | | | | | 1. Not at all sure 2. Somewhat unsure 3. Neither sure/unsure 4. Somewhat sure 5. Completely sure | |  |
| 326 | How sure are you that you could go to the health facility if your husband objected to your going? | | | | | | | | 1. Not at all sure 2. Somewhat unsure 3. Neither sure/unsure 4. Somewhat sure 5. Completely sure | |  |
| 327 | Do you feel confident to resolve a problem on your own? | | | | | | | | 1. Always  2. Sometimes  3. Not at all | |  |
| 328 | Do you feel like an important member of your household? | | | | | | | | 1. Always  2. Sometimes  3. Not at all | |  |
| 329 | Do you think the members of your household listen to you and respect your opinion? | | | | | | | | 1. Always  2. Sometimes  3. Not at all | |  |
